# Development and Validation of a Prediction Model for the Treatment Time of Deformational Head Shapes Using a Cranial Remolding Orthosis

**DOI:** 10.3390/children9030354

**Published:** 2022-03-04

**Authors:** Tiffany Graham, Jijia Wang

**Affiliations:** 1 Department of Prosthetics-Orthotics, University of Texas Southwestern Medical Center, 6011 Harry Hines Blvd, Dallas, TX 75390-9091, USA; 2 Department of Applied Clinical Research, University of Texas Southwestern Medical Center, 6011 Harry Hines Blvd, Dallas, TX 75390-9091, USA; Jijia.Wang@utsouthwestern.edu

**Keywords:** plagiocephaly, brachycephaly, asymmetrical brachycephaly, cranial remolding orthosis, helmet, treatment time

## Abstract

The cranial remolding orthosis (CRO) has been shown in previous studies to be an effective method of treatment for deformational head shapes. Many studies have shown younger infants achieve greater correction than older infants and generally have a shorter treatment duration. The goal of this study is to develop and validate a prediction equation for the maximum treatment time for deformational head shapes when utilizing a CRO. This retrospective study included subjects with deformational plagiocephaly (DP), deformational brachycephaly (DB), or deformational asymmetrical brachycephaly (DAB) who began CRO treatment between 3 and 18 months of gestational age. Prediction models were derived from 1250 subjects with DP, DB, and DAB and the validation used data from 210 different subjects. Actual treatment time was less than or equal to predicted treatment time in 85.19% (DP), 56.67% (DB), and 75.40% (DAB) of the cases when rounding the prediction up to the nearest month. The prediction equation has moderate accuracy for predicting the likely maximum amount of CRO treatment time for patients with DP, DB, and DAB and may be used clinically to give caregivers an estimated treatment duration for a patient who is indicated for a CRO, if treatment was initiated immediately.

## 1. Introduction

The infant skull can be deformed due to the application of external forces upon the skull during development [1,2]. The incidence of these deformational head shapes has dramatically increased since the American Academy of Pediatrics (AAP) began recommending supine sleep for infants and is now very common among infants in the United States [3,4]. While repositioning therapy can be an effective treatment, instances beyond a mild case are often treated with cranial remolding orthoses (CROs) [1,5,6].

Isolated deformational plagiocephaly (DP) describes the posterior lateral flattening of the skull and a subsequent bossing of the ipsilateral forehead. Risk factors for DP include multiple births, congenital anomalies, prematurity, male sex, and congenital muscular torticollis (CMT) [1,2,3,4,7]. Isolated deformational brachycephaly (DB) is characterized by a symmetric head shape that is wider than the norm. Deformational asymmetrical brachycephaly (DAB) has features of DB, but also has asymmetry [5].

A CRO is a custom-made Class II medical device intended to reshape an infant’s head by redirecting cranial growth [8]. It is made from a plaster cast or digital scan of an infant’s head. The CRO is designed to snugly fit over areas where the head does not need to grow and leave gaps over areas where skull growth is desired in order to direct cranial growth over time into a more normal shape [1].

In assessing and measuring the patient’s skull, measurements frequently include Cephalic Index (CI), Cranial Vault Asymmetry (CVA), and Cranial Vault Asymmetry Index (CVAI). CI is calculated by dividing the head width by head length and multiplying by 100, with values between 75 and 85% considered normal [9]. CVA is also referred to as the diagonal, oblique diagonal, or transcranial diagonal difference. It is defined as the difference between two diagonals from anterior lateral forehead to the opposite posterior lateral skull (frontozygomatic to opposite eurion [10], taken 30 degrees from midsagittal line) [11]. CVAI is a measurement of two-dimensional lateral cranial asymmetry, and calculated by the CVA divided by the longer diameter, multiplied by 100; it is evaluated independent of head size [2,9]. As CVAI is a proportion of deformity compared to the overall size of the head and this study measures infant ranging from 3 to 18 months of age, this study uses CVAI to measure cranial asymmetry instead of CVA. All measurements are taken at the level of the greater equator of the skull.

There are several factors that have been proven to influence the amount of treatment time a patient requires in a CRO and the results of the orthotic treatment. Starting age of treatment is a highly discussed factor in the literature. While there is not a strongly recommended starting or ending age of treatment, the Food and Drug Administration limits the use of CROs to infants between 3 and 18 months of age [8] and studies have shown earlier intervention is correlated with better outcomes [5,12,13,14]. In addition to infant age, the severity of deformation and desired change are also factors which influence length of treatment. According to Kunz et al., “The degree of asymmetry reduction decreased with increased age at the start of treatment, whereas the treatment length increased [15].” Kunz et al. also found that required treatment length is more closely related to gestational age than birth age [16], which indicates that treatment initiation age should be calculated with considerations taken for prematurity. Weersma, et al. noted that there are large individual variations in the rate of change of CVAI and CI among subjects, even within the same treatment center. This could be due to individual growth patterns, variations in clinical methods of the treating clinicians, or variations in patient compliance with the orthotic wear schedule, among other factors [5].

Previous studies by this cohort have shown correlations between the age of the infant at the start of CRO treatment, along with head shape severity measurements, and treatment duration. Specifically, infants with more severe measurements and older infants tend to have longer treatment times. Likewise, for DP, it was found that younger infants who present with less severe CVAI and no torticollis typically have more positive treatment outcomes while infants with torticollis seem to have extended treatment times compared to those without torticollis [17].

Clinically, it appears several factors influence treatment time with a CRO but to the best of our knowledge, the estimation of CRO treatment duration remains poorly developed. Commonly, when a caregiver requests and estimated treatment time from a practitioner, the clinician must answer the question based on their past clinical experience. A treatment time prediction model would aid clinicians in answering this common caregiver’s question.

This study is a retrospective chart study which attempts to derive and validate prediction models for the estimated maximum CRO treatment duration for patients with a diagnosis of DP, DB, or DAB. The input for these models includes the age of the infant at the initiation of treatment (corrected for prematurity), initial CVAI and CI, clinician-desired CVAI and CI to discharge from treatment, as well as the presence or absence of torticollis and prematurity. It produces a predicted maximum CRO treatment duration.

## 2. Materials and Methods

The first goal of this study was to generate a prediction model for the maximum CRO treatment duration for subjects with DP, DB, or DAB. The charts reviewed for derivation of the prediction models were from Level 4 Prosthetics & Orthotics (Restore POC) the Addison, TX location, as well as two locations in San Antonio, TX, USA. These three offices used the same methods to evaluate patients, fabricate orthoses, and train their clinicians in the same manner. All offices used the STARband brand CRO (Orthomerica Products, Inc, Orlando, Florida). The prediction models were validated using the data from patients at the University of Texas Health Science Center San Antonio (UTHSCSA) located in San Antonio, TX, USA. The treatment protocols for both companies included fitting the CRO within 2 weeks of a fabrication scan, then seeing the patient every 1–4 weeks (depending on growth rate) for adjustments to the CRO to direct skull growth throughout treatment. Infants were discharged from orthotic treatment according to the protocol used at the treatment clinics, which included parental and treating practitioner satisfaction or reaching a head shape that was considered either mild or within normal limits (CVAI less than 6.25 and a CI less than 90%). Other discharge reasons included that the patient’s growth rate had become too slow to notice continued change with orthotic treatment (usually occurring in older infants due to the normal slowing of growth with age) and the infant outgrowing the CRO. For the derivation of the predictive models, only infants who completed orthotic treatment (according to the treating practitioner) were included. For the validation of the models, any patient at UTHSCSA who had subsequent scans, met the inclusion criteria, and were documented to be compliant with orthotic wear were included.

IRB approval for this study was obtained through the University of Texas Southwestern Medical Center’s institutional review board (DP Study #STU 032016-077, DB Study #STU-2018-0093, and DAB Study #STU 022017-046). As a part of the IRB approval, infant ages were required to be rounded to the nearest half month (for DB and DAB patients), or whole month (for DP patients). Due to the retrospective design of the study, no direct recruitment of subjects occurred. Any necessary recruitment or consenting was done by Level 4 (Restore POC) or UTHSCSA prior to study, in that the subjects agreed to receive treatment at these clinics.

The data collected included corrected treatment age of the subjects at the initiation of treatment, CVAI and CI at the initiation of treatment, CVAI and CI at the end of treatment, the presence or absence of torticollis, and the presence or absence of prematurity. Prematurity was defined as less than or equal to 37 weeks gestation. Similarly, the presence or absence of torticollis was recorded to indicate a diagnosis of torticollis or documented resistance through range of motion. Treatment age was calculated by recording the postpartum age of the infant and subtracting the number of weeks of prematurity (if the infant was born at 37 weeks gestation or earlier), then rounding the age to the nearest half month (DB and DAB) or whole month (DP). The amount of correction was defined as the difference in CVAI and/or CI (as applicable for each head shape) from the start to end of treatment.

The scanner used was the STARscanner by Orthomerica (Orlando, FL, USA). The STARscanner is a laser data acquisition system which collects the subject’s head shape in under two seconds. The head shape was recorded using the Yeti Shape Builder software. This image was uploaded into the Cranial Comparison Utility (CCU) software for measurements. CCU produces a STARscan report which was then placed in patient records. Both Yeti Shape Builder and CCU are manufactured by Vorum Research Corporation (Vancouver, BC, Canada).

To be included in the model derivation, subjects must have been diagnosed with a deformational head shape and referred to Level 4 Prosthetic and Orthotic (Restore POC) clinics for treatment. Subjects must have a starting CVAI and CI value in accordance with their head shape: For DP, subjects must have a starting CVAI value greater than or equal to 3.5 and CI less than 90%. For DB, subjects must have a starting CVAI less than 3.5 and CI greater than or equal to 90%. For DAB, subjects must have an initial CVAI greater than or equal to 3.5 and CI greater than or equal to 90%. Any other starting value for CVAI or CI was excluded. The subjects also need to have started treatment between 3 and 18 months of postpartum age. Subjects were excluded if they had any other positional head deformities such as scaphocephaly or asymmetrical scaphocephaly. Subjects with synostotic head shapes or significant comorbidities other than prematurity and torticollis were excluded from the study. Subjects with torticollis or who were premature were included because they are a significant portion of the patient population. Subjects who dropped out of treatment prior to completion or subjects with documented orthotic wear non-compliance were excluded from the design.

The second goal of this study was to validate the derived prediction models using a different data set. The same inclusion and exclusion criteria were used for subjects from UTHSCSA for validation of the prediction models. The subjects must have been diagnosed with a deformational head shape and referred to UTHSCSA for treatment. In addition, subjects were included if they have more than one scan on file, even if they withdrew from treatment later despite practitioner recommendations. Since these predictive models need exact starting and ending head shape measurements to be manually inputted, measurements from all UTHSCSA compliant infants were used in the retrospective validation. The predictive models were tested using inputs from the actual head shape measurement changes seen by UTHSCSA patients over the course of their treatment. The predicted treatment time for UTHCSCA to reach their actual end of treatment head shape measurements was then compared to the actual treatment time UTHCSCA patients experienced. For predictive use of the models in the future, clinicians should input the patient’s current head shape measurements and demographics, then input their head shape measurement treatment goals in order to have estimated maximum treatment time outputted by the models.

All statistical analyses were conducted using statistical software SAS 9.4 (SAS Institute Inc, Cary, NC). The descriptive statistics (mean, standard deviation, frequency, and percentage) were used to summarize the demographic and clinical variables. The multiple linear regressions were used to build the prediction equation for DP, DB, and DAB, respectively, using the patients’ data from Level 4 Prosthetic and Orthotic (Restore POC) clinics, followed by validation of the models using the patients’ data from UTHSCSA to compare the model’s predicted treatment time with the infant’s actual treatment time. The level of significance was set at 5%.

The third goal of this study was to create a potentially more accurate prediction model using combined data sets. Therefore, once the accuracy of the initial prediction models was examined, the process was repeated with the combined data from Level 4 (Restore OPC) and UTHSCSA. Final prediction models were derived, which can be validated in the future.

## 3. Results

To create the prediction models, treatment and evaluative data from subjects treated at Level 4 Prosthetics & Orthotics (Restore POC) were retrospectively recorded. In total, 2423 charts from January 2013 to June 2017 yielded 497 included DP subjects, 2977 charts from January 2013 to June 2019 yielded 253 included DB subjects, and 2104 charts from January 2013 to June 2017 yielded 500 included DAB subjects. These 1250 subjects’ descriptive statistics are shown in Table 1 and the derived prediction equations for maximum CRO treatment time are found in Table 2.

The inputs for the prediction equation for DP includes the corrected age at the initiation of treatment, the starting CVAI, the difference between starting and ending CVAI, the presence of torticollis, and presence of prematurity. The derived prediction equation for DB uses starting and ending CI instead of CVAI, and the derived prediction for DAB uses both CVAI and CI. The reason for excluding CI in the prediction time for DP and excluding CVAI in the prediction for DB is that clinically, these measurements were not being actively treated for these head shapes. The correction of DP focuses on the correction of CVAI and the correction of DB focuses on changes in CI. The correction of DAB requires correction of both CI and CVAI measurements.

Charts from 352 patients at UTHSCSA’s cranial remolding program of infants who began treatment between November 2017 and January 2020 were retrospectively reviewed. The prediction equation for DP was tested using 54 of these subjects, the prediction equation for DB was tested using 30 of these subjects, and the prediction equation for DAB was tested using 126 of these subjects. The remaining 142 charts were excluded due to deformational head shapes that did not fit in our categories, craniosynostosis, other comorbidities that impact growth, non-compliance, or had treatment outside of the IRB approved timeframe. The 210 subjects included in the study covered a wide range of ages and severities (Table 1). Of the 210 subjects, 118 presented with torticollis and 66 were born premature. All subjects began their treatment between the corrected ages of 3–18 months.

**Figure 1:**
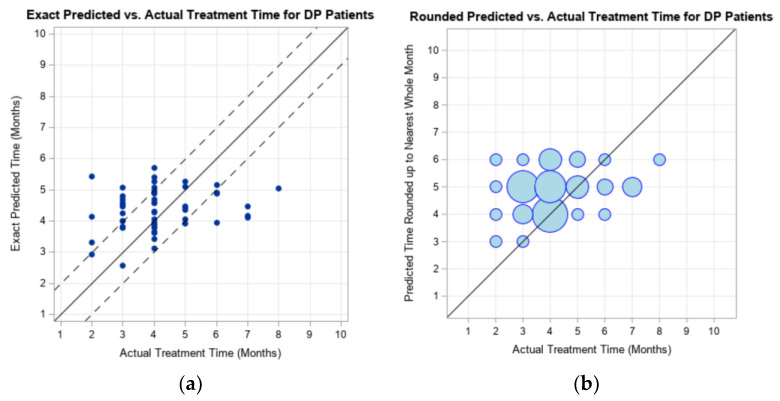
Graphical representation of the exact model prediction (**a**) and rounded model prediction (**b**) of maximum treatment duration compared to the actual CRO treatment duration experienced the 54 UTHSCSA subjects with deformational plagiocephaly (DP). Dotted lines represent ± 1 month of actual treatment duration.

Figure 1 gives graphical representations of the predicted maximum treatment time generated by the model versus the actual clinical treatment time experienced by the 54 infants at UTHSCSA which had DP. Predictions between the dotted lines on Figure 1a represents a prediction that is within ±1 month of the actual treatment time experienced by the infant. Predictions which are on or above the solid line in Figure 1a represent “accurate” maximum treatment times, as the model’s prediction was equal to or greater than the treatment time experienced by the infant. In these cases, the caregiver would have been pleased to complete treatment in the predicted timeframe or less.

The accuracy of the prediction model output was evaluated using the exact maximum prediction time and the maximum prediction time which has been rounded up to the nearest whole month. When the prediction model output was rounded up, 85.19% of the subjects had an overestimation of treatment time, when compared to the actual duration of treatment (Figure 1b and Table 3).

**Figure 2:**
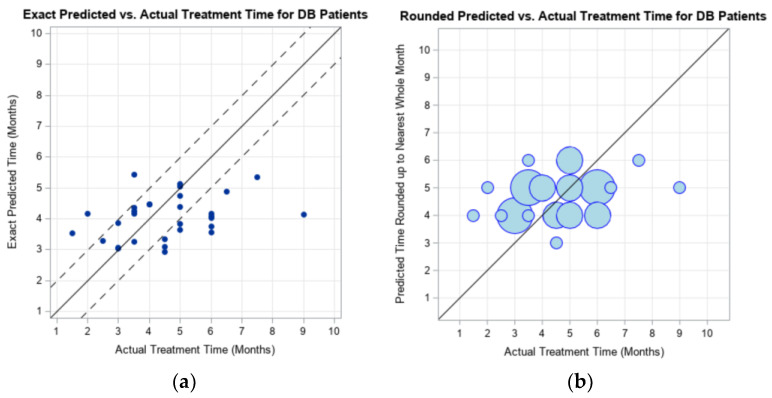
Graphical representation of the exact model prediction (**a**) and rounded model prediction (**b**) of maximum treatment duration compared to the actual CRO treatment duration experienced the 30 UTHSCSA subjects with deformational brachycephaly (DB). Dotted lines represent ± 1 month of actual treatment duration.

The evaluation forDB was conducted similar to the assessment for DP. The prediction model output was compared to the actual treatment time that 30 UTHSCSA subjects experienced (Figure 2). The prediction was evaluated using three different model output results (depending on rounding of numbers): the exact prediction, the prediction rounded to the nearest whole month, and the prediction rounded to the nearest half month. When the model output was rounded to the nearest whole month, 56.67% of the subjects had an overestimation of treatment time, when compared to the actual duration of treatment (Table 3). Figure 2 is the graphical representation of the clinical treatment time reported by UTHSCSA for these subjects.

**Figure 3:**
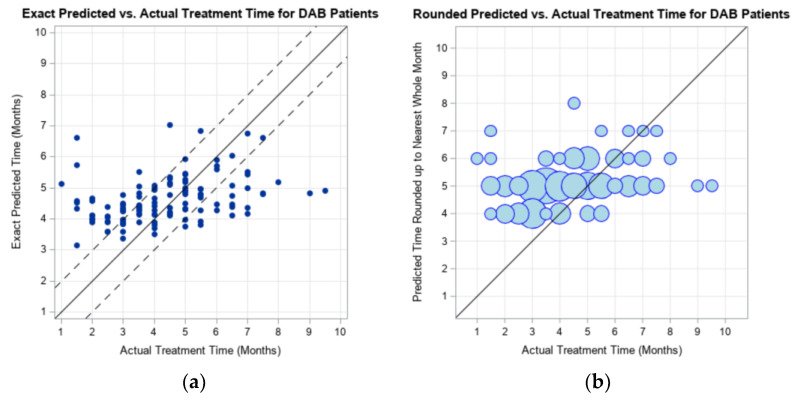
Graphical representation of the exact model prediction (**a**) and rounded model prediction (**b**) of maximum treatment duration compared to the actual CRO treatment duration experienced the 126 UTHSCSA subjects with deformational asymmetrical brachycephaly (DAB). Dotted lines represent ± 1 month of actual treatment duration.

Evaluation of the subjects with DAB was completed through the same process as described for those with DB. The prediction model output was compared to the actual treatment time that the 126 UTHSCSA subjects experienced. When the model output was rounded to the nearest whole month, 75.40% of the subjects had an overestimation of treatment time, when compared to the actual duration of treatment. Figure 3 is the graphical representation of the clinical treatment time reported by UTHSCSA for subjects with DAB.

If the predicted treatment time is greater than or equal to the actual treatment time reported in the patient chart, we concluded our model has accurate prediction ability for the longest estimated treatment duration. How often the treatment time prediction was within ± 1 month of the actual treatment time was also examined. The results for each head shape are listed in Table 3.

The DP model was able to predict the actual treatment time within ±1 month of actual treatment time in 55.56% of cases. When the difference between the actual treatment time the infant experienced and the model’s predicted treatment time were averaged together, the models were found to generally over-estimate treatment time by 6 days (SD = 40 days). The DB model was able to predict the actual treatment time within ±1 month of actual treatment time in 46.67% of cases. The model had an overall under-estimation of treatment time (compared to actual treatment time) by 15 days (SD = 47 days). The DAB model was able to predict the actual treatment time within ±1 month of actual treatment time in 50.79% of cases. The model, on average, over-estimated treatment time by 10 days (SD = 48 days).

After this validation attempt, the prediction models were reanalyzed by repeating the derivation process using data from both facilities. The resulting updated, non-validated prediction models are shown in Table 4.

## 4. Discussion

When the output of the DP prediction model was rounded up to the nearest whole month, the prediction model correctly predicted the maximum treatment duration in 85.19% of the UTHSCSA cohort. Similarly, this was true in 56.67% of the cases of DB and 75.40% of cases for DAB. These percentages represent the patient completing treatment equal to or faster than predicted time, which clinicians and caregivers would consider to be successful. It is likely that the reason for the lower accuracy for DB is due to the lower number of subjects used in both the derivation and in the validation compared to DP and DAB. With additional DB subjects, it is probable that this prediction model would become more accurate. The accuracy of the DP prediction is higher than that of the DAB prediction likely because the treatment of DP focuses on only adjusting one cranial measurement (CVAI), which typically involves directing growth into the flattened posterior quadrant and contralateral anterior quadrant of the infant’s skull while keeping the CI relatively stable throughout treatment. The treatment of DAB involves changing more of the skull’s shape than DP as it attempts to lower both CI and CVAI measurements throughout the treatment process.

The average predictions varied from the actual treatment time by −15 to +10 days. Clinically, this is meaningful and valuable. The ability to predict a maximum expected length of treatment for patients with DP, DB, and DAB has benefits over relying solely on a practitioner’s experience to provide an estimation. An accurate expected treatment length can provide realistic expectations to the caretakers of the infant and set them up for successful treatment. The parents and caretakers significantly influence the course and success of treatment through properly donning the CRO, ensuring the brace stays on the prescribed 23 h/day, inspecting the infant’s skin, and attending regular follow up visits with the treating clinician for CRO adjustments. Caretakers of infants needing CRO treatment usually have many questions regarding treatment, a prediction model will allow practitioners to more accurately answer these questions, thus improving parental and caretaker perception of treatment.

There are several limitations to this study that should be noted. One limitation is the assumption of patient compliance to the 23 h per day wear schedule. Subjects were excluded if there was evidence of non-compliance documented by the treating practitioner or obvious global growth in successive scans. Deviation from the recommended wear schedule could influence results as the orthosis may not capture the full growth of the infant’s head. In addition to this, the model also does not account for the differences in individual growth rate of the infants. Infants will grow at different rates in a given amount of time which will affect the rate that the deformational head shape is corrected because growth is the driving factor of correction [2]. It should be noted that although the predictions were relatively accurate on average, individual results of the predictions varied significantly, some having as much as a 5-month variance between predicted treatment time and actual treatment time (DP range −2.94 to 3.43 months, DB range −4.85 to 2.17 months, and DAB range −4.58 to 5.12 months) and the R-squared values of the prediction models are low (DP R^2^ = 0.30, DB R^2^ = 0.20, and DAB R^2^ = 0.19). However, these models are not intended to predict exact treatment duration, but are intended to predict maximum likely treatment duration. Another limitation is that individuals with developmental delays and other syndromes which could affect growth were excluded from the study; however, there may have been some infants included who had undiagnosed conditions at the time of CRO treatment. The presence of these conditions can affect the growth rate and consequently the rate of correction of deformational head shape. These equations should be used as a tool to educate caregivers of probable treatment time, should their infant start treatment at their current age and with their current head shape. If treatment is not initiated at that time, the changes in the shape of the head over time could significantly impact the infant’s overall treatment time with a CRO.

It is also important to note that these maximum treatment time prediction models do not necessarily reflect the average treatment duration found for infants in the examined data sets. The prediction is intended only to be used to aid discussing caregiver expectations. These models only apply if their child has no comorbidities which may affect their cranial growth, if the infant wears the orthosis 23 h per day, if the infant starts CRO treatment at their current head shape measurements, if the infant begins treatment at their current age, and if a practitioner inputs the desired treatment head shape measurement goals. If the infant’s inputted information or practitioner’s treatment goals were to change, this would change the maximum treatment time estimate.

Additionally, this prediction equation estimates the maximum time between fabrication of the orthosis and the completion of treatment. This means the measurements at the time of fabrication are used, not the measurements of the infant at the time of the fitting of the orthosis. In the case of all studied treating facilities, the practitioner protocol was to fit a CRO 1.5–2 weeks after the fabrication scan. Since the protocol of treating facilities did not include taking an additional scan at the time of the fitting, it is unknown if the infants head shape changed between the fabrication scan and the fitting of the CRO. When applying these equations to treatment protocols at other facilities, the estimation may be skewed if the time between fabrication and fitting of the CRO varies from this 1.5–2 weeks.

Although the results of this study are promising, there is still future work to be done. In the initial derivation of the model, the difference in CVAI was found to be negatively correlated with overall treatment time for DP and DAB, and the difference in CI was found to be negatively correlated with overall treatment time with DB. That being said, the difference in CVAI was also not found to be significant when determining the treatment time for DAB (*p* = 0.1722) nor the difference in CI for the prediction of the treatment time for DB (*p* = 0.7795). In both these cases, the starting CVAI and CI were found to be significant (*p* < 0.0001, for both). The finding that the difference in CVAI and CI does not influence treatment time significantly is inconsistent with current literature and clinical empirical findings, which show that correction is related to treatment time, with infants achieving more correction the longer they wear their CRO [5,12]. Further investigation will be conducted to reveal the underlying reason for this unexpected result and it is possible that a larger number of subjects would change this result. Additionally, analyses should be done to narrow down the projected treatment time and improve the accuracy of the prediction model. The prediction equation was derived and validated using initial corrected age rounded to the nearest whole month for DP but by the nearest half month for DB and DAB due to IRB constraints which may have affected accuracy. Ideally, exact treatment durations would have been used.

The updated prediction equations shown in Table 4 have not been validated at this time. It is hypothesized that by including more data from additional facilities, the treatment time predictions will become more accurate. Of note, all facilities examine in this study use the STARband brand CRO. Further investigations are needed to determine if the predictions hold true for different brands of CROs and different treatment facilities. Several derivations of this prediction model may be needed to improve accuracy. The clinical goal is to create a formula that would be distributed to practitioners, allowing them to input necessary patient information and yield an accurate expected length of treatment to share with parents in real-time.

## 5. Conclusions

The conducted pilot study creates and attempts to validate a prediction model for the maximum treatment time for patients with deformational head shapes using a CRO. The accuracy of each prediction model indicated there is predictability for treatment time for deformational head shapes while using a CRO. The tested model was shown to have an accuracy of 57–85% when the maximum treatment time prediction was rounded up to the nearest month. The presented models have the potential to provide clinical benefits for practitioners to determine the estimated maximum amount of time a patient will require treatment, assuming they are compliant with the 23 hour per day wear protocol and have a normal growth pattern. Further investigation with larger sample sizes is needed to improve the accuracy of the models and future investigations should address the universality of the models by examining outcomes of infants at several different treatment centers and for those who use a different brand of cranial orthosis.

## Figures and Tables

**Table 1:** Descriptive statistics of the data sets gathered from Level 4 (*n* = 497 DP, *n* = 500 DAB, and *n* = 253 DB) and UTHSCSA (*n* = 54 DP, *n* = 126 DAB, and *n* = 30 DB).

Demographic	Clinic	Measurement	Deformational Plagiocephaly (DP)	Deformational Brachycephaly (DB)	Deformational Asymmetrical Brachycephaly (DAB)
Corrected Age at Start (months)	Level 4	Mean ± SD Range	6.06 ± 2.04 2 to 17	6.14 ± 1.99 2 to 13	5.95 ± 1.97 2 to 17
	UTHSCSA	Mean ± SD Range	6.57 ± 2.08 4 to 14	6.88 ± 2.54 4 to 17	5.87 ± 2.04 3 to 18
Premature Subjects	Level 4	Count	142	71	155
	UTHSCSA	Count	12	6	48
Subjects with Torticollis	Level 4	Count	299	32	201
	UTHSCSA	Count	44	5	69
Initial CVAI	Level 4	Mean ± SD Range	7.91 ± 2.29 3.5 to 16.10	(all below 3.5)	6.85 ± 2.08 3.5 to 14.70
	UTHSCSA	Mean ± SD Range	8.45 ± 1.95 4.55 to 13.13	(all below 3.5)	7.06 ± 2.08 3.57 to 13.28
Final CVAI	Level 4	Mean ± SD Range	3.36 ± 1.40 0.10 to 10.10	(all below 3.5)	2.90 ± 1.40 0.10 to 10.70
	UTHSCSA	Mean ± SD Range	3.59 ± 1.33 0.82 to 9.90	(all below 3.5)	2.99 ± 1.52 0.00 to 8.22
Initial CI	Level 4	Mean ± SD Range	(all below 90%)	97.70% ± 3.44% 90.10% to 107.70%	94.67% ± 3.29% 90% to 109.80%
	UTHSCSA	Mean ± SD Range	(all below 90%)	96.31% ± 2.98% 90.50% to 100.90%	95.07% ± 3.73% 90.10% to 108.50%
Final CI	Level 4	Mean ± SD Range	(all below 90%)	91.58% ± 2.57% 83.60% to 98.10%	90.54% ± 2.39% 81.30% to 102.80%
	UTHSCSA	Mean ± SD Range	(all below 90%)	91.10% ± 2.60% 85.90% to 96.70%	90.02% ± 2.71% 84.60% to 102.70%
Actual Treatment Time (months)	Level 4	Mean ± SD Range	3.94 ± 1.63 1 to 11	4.19 ± 1.59 1 to 10	4.37 ± 1.68 1 to 11
	UTHSCSA	Mean ± SD Range	4.13 ± 1.33 2 to 8	4.57 ± 1.63 1.5 to 9	4.31 ± 1.71 1 to 9.5

**Table 2:** Treatment time prediction equations derived from 497 deformational plagiocephaly subjects, 253 deformational brachycephaly subjects, and 500 deformational asymmetrical brachycephaly subjects.

Head Shape	Treatment Time Prediction Equation
Deformational Plagiocephaly (DP) ^1^	$\begin{array}{c} Treatment\;time=-0.341+0.210*\left(Corrected\;Age\;at\;Start\right)+0.440*Initial\;CVAI-\\ 0.164*Difference\;in\;CVAI+0.415*Torticollis+0.085*Prematurity. \end{array}$
Deformational Brachycephaly (DB) ^1^	$\begin{array}{c} Treatment\;time=-16.558+0.169*\left(Corrected\;Age\;at\;Start\right)+0.203*Initial\;CI-\\ 0.016*Difference\;in\;CI-0.215*Torticollis-0.143*Prematurity. \end{array}$
Deformational Asymmetrical Brachycephaly (DAB) ^1^	$\begin{array}{c} Treatment\;time=-7.893+0.208*\left(Corrected\;Age\;at\;Start\right)+0.094*Initial\;CI+\\ 0.117*Difference\;in\;CI+0.268*Initial\;CVAI-0.087*Difference\;in\;CVAI+0.376*\\ Torticollis+0.021*Prematurity. \end{array}$

^1^ Input terms: *Treatment time:* The estimated maximum time the patient will need to wear their orthosis to achieve the desired head shape measurement goals. Time is given in months, and is the time between the fabrication scan and the expected treatment discharge date. *Corrected Age at Start:* Input the patient’s age at the time of orthosis fabrication, corrected for prematurity, in months. *Initial CVAI:* Input the patient’s current cranial vault asymmetry index (CVAI). *For example, a CVAI of 8.5 should be inputted as “8.5”. Initial CI:* Input the patient’s current cephalic index (CI). *For example, a CI of 95% should be inputted as “95”. Difference in CVAI:* Input the patient’s current CVAI minus their treatment goal CVAI. *For example, if the patient has a current CVAI of 8.5 and the treatment goal is a CVAI of 3, this should be inputted as “5.5”. Difference in CI:* Input the patient’s current cephalic index (CI). *For example, if the patient has a current CI of 95% and the treatment goal is a CI of 88%, this should be inputted as “7”. Torticollis:* Input either “0” or “1” to indicate if the patient does not have torticollis (0) or has torticollis (1). *Prematurity:* Input either “0” or “1” to indicate if the patient is not premature (0) or is premature (1).

**Table 3:** Accuracy of the derived prediction equation for treatment times for each head shape.

Prediction Model Accuracy		
**Prediction Time (rounded up to the nearest whole month)**	**Predicted Time ≥ Actual Treatment time**	
	**Frequency**	**Percentage**
Deformational Plagiocephaly (DP)	46 out of 54	85.19%
Deformational Brachycephaly (DB)	17 out of 30	56.67%
Deformational Asymmetrical Brachycephaly (DAB)	95 out of 126	75.40%
**Prediction Time (rounded up to the nearest half month)**	**Predicted Time ≥ Actual Treatment time**	
	**Frequency**	**Percentage**
Deformational Brachycephaly (DB)	16 out of 30	53.33%
Deformational Asymmetrical Brachycephaly (DAB)	93 out of 126	73.81%
Prediction Time within ± 1 month of actual time	Frequency	Percentage
Deformational Plagiocephaly (DP)	30 out of 54	55.56%
Deformational Brachycephaly (DB)	14 out of 30	46.67%
Deformational Asymmetrical Brachycephaly (DAB)	64 out of 126	50.79%

**Table 4:** Treatment time prediction equations derived from 551 deformational plagiocephaly subjects, 283 deformational brachycephaly subjects, and 626 deformational asymmetrical brachycephaly subjects; spanning two different companies and four treatment facilities.

Head Shape	*Updated* Treatment Time Prediction Equation
Deformational Plagiocephaly (DP) ^1^	$\begin{array}{c} Treatment\;time=-0.127+0.189*\left(Corrected\;Age\;at\;Start\right)+0.442*Initial\;CVAI-\\ 0.176*Difference\;in\;CVAI+0.345*Torticollis-0.006*Prematurity. \end{array}$
Deformational Brachycephaly (DB) ^1^	$\begin{array}{c} Treatment\;time=-16.167+0.149*\left(Corrected\;Age\;at\;Start\right)+0.201*Initial\;CI-\\ 0.015*Difference\;in\;CI-0.118*Torticollis-0.110*Prematurity. \end{array}$
Deformational Asymmetrical Brachycephaly (DAB) ^1^	$\begin{array}{c} Treatment\;time=-6.543+0.221*\left(Corrected\;Age\;at\;Start\right)+0.078*Initial\;CI+\\ 0.156*Difference\;in\;CI+0.189*Initial\;CVAI+0.020*Difference\;in\;CVAI+0.300*\\ Torticollis+0.071*Prematurity. \end{array}$

^1^ Input terms: *Treatment time:* The estimated maximum time the patient will need to wear their orthosis to achieve the desired head shape measurement goals. Time is given in months, and is the time between the fabrication scan and the expected treatment discharge date. *Corrected Age at Start:* Input the patient’s age at the time of orthosis fabrication, corrected for prematurity, in months. *Initial CVAI:* Input the patient’s current cranial vault asymmetry index (CVAI). *For example, a CVAI of 8.5 should be inputted as “8.5”. Initial CI:* Input the patient’s current cephalic index (CI). *For example, a CI of 95% should be inputted as “95”. Difference in CVAI:* Input the patient’s current CVAI minus their treatment goal CVAI. *For example, if the patient has a current CVAI of 8.5 and the treatment goal is a CVAI of 3, this should be inputted as “5.5”. Difference in CI:* Input the patient’s current cephalic index (CI). *For example, if the patient has a current CI of 95% and the treatment goal is a CI of 88%, this should be inputted as “7”. Torticollis:* Input either “0” or “1” to indicate if the patient does not have torticollis (0) or has torticollis (1). *Prematurity:* Input either “0” or “1” to indicate if the patient is not premature (0) or is premature (1).

## Data Availability

Due to limitations in the data use agreements between the University of Texas Southwestern Medical Center and both Level 4 Prosthetics & Orthotics as well as the University of Texas Health Science Center San Antonio, raw data cannot be shared publicly.
